# Physiological and transcriptomic analysis of salt tolerant *Glaux maritima* grown under high saline condition

**DOI:** 10.3389/fpls.2023.1173191

**Published:** 2023-08-29

**Authors:** Rui Gu, Zhi Qiang Wan, Fang Tang, Xue Ting Liu, Yan ting Yang, Feng ling Shi

**Affiliations:** ^1^ Key Laboratory of Grassland Resources of Ministry of Education, College of Grassland, Resources and Environment, Inner Mongolia Agricultural University, Hohhot, Inner Mongolia, China; ^2^ College of Geographical Science, Inner Mongolia Normal University, Hohhot, China

**Keywords:** *Glaux maritima*, salt gland, physiology, transcriptome analysis, DEGs

## Abstract

Land salinization considerably limits crop production. Biological improvement of saline and alkaline land is an important way to achieve efficient land use. It is crucial to study the salt tolerance of halophyte resources in order to explore and improve plant resources through biological improvement. *Glaux maritima* is a mesophyte halophyte with strong salt tolerance. In this study, we conducted research on the salt tolerance mechanism of *G. maritima* through phenotypic, physiological, and transcriptomic aspects. The results indicate that leaf cross-sections revealed that *G. maritima* has a salt gland tissue composed of stalk, collecting, and secretory cells, which are trapped in epidermal cells. At the physiological level, the maximum salt tolerance threshold of *G. maritima* leaves was 600 mM/L. At this concentration, proline content, relative conductivity, and superoxide dismutase (SOD), peroxidase (POD), and catalase (CAT) enzyme activities were maximum. At the transcriptional level, transcriptome data of three experimental groups (N0: 0 mM/L, N3: 600 mM/L, and N4: 800 mM/L) were analyzed, and six essential genes related to proline synthesis and five essential genes related to SOD and CAT enzyme activities were identified. Two genes involved in CAT enzyme activity were also found to play an important role in the MAPK signaling pathway. Trend analysis revealed that the MAPK signaling regulation (37 differentially expressed genes (DEGs)), phytohormone regulation (48 DEGs), glutathione metabolism (8 DEGs), flavonoid and flavonoid biosynthesis (2DEGs), and flavonoid biosynthesis (24 DEGs) pathways played important roles in regulating the salt tolerance of *G. maritima*. These findings provide valuable information for further studies on the functional characteristics of *G. maritima* in response to abiotic stress and may contribute to salt resistance breeding of fodder crops for cultivation in saline alkali land.

## Introduction

The salinization of soil is a major abiotic stressor that inhibits plant development. It is estimated that 831 million hectares, or around 6% of the world’s total land area, are ruined by salt and cannot be utilized for agricultural (Shrivastava and Kumar, 2015; Kuang et al., 2019; Safdar et al., 2019). Intensifying salinization of soils is a result of both human activity and climate change, and this has devastating effects on plant development and agricultural yields in salinized regions (Munns et al., 2019). Using and improving arid and salty soil is greatly facilitated by halophytes. Increased land-use efficiency, reduced soil erosion, and remediation of soil salinity are all possible due to halophytes, which can develop regularly despite harsh environmental circumstances including high salt and drought. Studies have shown that wild species of the 5 genera *Artemisia vulgaris* L.*, Artemisia absinthium* L.*, Chenopodium album* L.*, Sanguisorba minor* Scop. and *Salsola komarovii* Iljin. can not only grow normally on land affected by different degrees of salinization, but can also be used to remediate soil salinity and heavy metals (Rebhi et al., 2019; Calone et al., 2021; Trofimova et al., 2012; Walker et al., 2014; Saddhe et al., 2020; Tlili et al., 2020).

Adaptation of halophytes to salt stress is reflected in their morphology and physiology. Halophytes often have a unique salt-tolerant structure that resists salt damage. Studies have shown that the unique structures of salt-secreting plants are mainly salt glands and salt vesicles (Wu et al., 2020). Salt glands are commonly found in monocotyledons, such as *Aeluropus, Sporobolus, Spartina*, and *Zoysia* of the Gramineae family, and flowering dicotyledons in the desert and coastal habitats, such as *Avicenia, Tamarix, Reaumuria* Linn., and *Limonium* Mill. These plants contain multicellular salt glands with different structures and cell numbers (Zhou et al., 2001; Wilson et al., 2017). Salt vesicles are mainly found in *Atriplex, Chenopodium, and Salsola* (Zhou et al., 2001). They are salt-secreting tissues that store salt first and discharge it after the salt vesicles mature and rupture. These tissues are composed of vesicle cells (EBCs), stalk cells (SCs), and epidermal cells (ECs) (Shabala et al., 2014). Physiologically, halophyte seedlings resist salt damage by the regulation of ionic, osmotic, and oxidative systems (Yuan et al., 2015). After salt stress, plant membrane lipid peroxidation decomposes into malondialdehyde, which destroys the structure and function of the plasma membranes, changes the permeability of the membrane, and affects the normal physiological and biochemical reactions of plants. Simultaneously, plant cytoplasm accumulates large amounts of proline, maintains intracellular homeostasis, and reduces ionic toxicity (Shabala et al., 2014). Antioxidants such as carotenoids and glutathione, which are produced by plants under oxidative stress, can trigger a large amount of antioxidants, especially ascorbic acid and glutathione. Plants respond to potentially damaging oxidative stress by turning on redox regulating enzymes like glutathione reductase (GR) and antioxidant enzyme systems including superoxide dismutase (SOD), and ascorbic acid peroxidase (APX), and catalase (CAT), among others (Yuan et al., 2012). In a study of the salt-tolerant growth strategy of the halophyte *Salicornia europaea*, it was found that, after salt stress, while biomass and chlorophyll content decrease, proline content and peroxidase activity increase (Ghanem et al., 2021). In saline-alkali lands, halophytes balance the osmotic pressure inside and outside the cells through their unique salt glands or salt vesicles. At the same time, the content of physiological and biochemical substances in the plant changes, affecting the osmotic pressure inside and outside plant cells and the permeability of the cell membrane, enabling halophytes to maintain normal growth in saline environments.

In order to discover the genes responsible for salt tolerance in halophytes, high-throughput transcriptome sequencing has been frequently employed. Zhao et al. (Zhao et al., 2014). mapped the transcriptome of *Salicornia paniculata* (Chenopodiaceae) and discovered that genes involved in glucanase, 6-phosphate dehydrogenase, aldehyde dehydrogenase, ethylene response factor, MYB, WRKY, and bZIP transcription factor, citrate synthase, and protein kinase play a significant role in plant salt tolerance under 600 mM NaCl stress. Transcriptome analysis of *Zoysia japonica* exposed to salt stress using RNA-seq technology has also been performed. The majority of DEGs show enrichment in GO functional categories including stimulation and stress responses (Xie et al., 2015). Transcriptome analysis of *Suaeda glauca* under salt stress showed that differential genes are significantly enriched in functional clusters, such as signal transduction, transporters, cell wall, and growth (Jin et al., 2016). Analysis of transcriptomes of *S. paniculata* under salt stress revealed the expression of the DNA damage repair-related genes *hcrev1* and *hcrev3* induced by salt stress (Du et al., 2017).

Inner Mongolia is located in northern China, which is a typical inland area and the climate is drought and less rain. As an important agricultural and animal husbandry crop producing area in China, the planting mode is mainly irrigation, leading to an increasing degree of secondary salinization in the soil year by year. There is an urgent need to explore salt tolerant mesophytes that are suitable for the ecological environment of the region and cultivate agricultural and pastoral crops with good salt tolerance. *Glaux maritima* L. is a plant of *Glaux* Linn. in *Primulaceae*, and is a mesophytic halophyte. Widely distributed in the western and northern regions of China. Although previous studies have conducted preliminary studies on the salt tolerance of *G. maritima* from aspects such as ion content and the ecology of community microorganisms under salt stress, there are still few reports on the salt tolerance adaptation strategies and molecular mechanisms of *G. maritima*. (Rozema, 1975; Rozema and Sminia, 1977; Yamamoto et al., 2020; Yamamoto et al., 2021). Therefore, this study conducted research on the morphological structure, physiological metabolism and Transcriptome of *G. maritima*. seedlings under varying salt concentrations. To provide theoretical reference for later salt tolerant gene mining and provide new resource support for improving salt tolerance of agricultural and livestock crops, and provides guidance for improving the salt tolerance of crops and their cultivation in saline-alkali areas.

## Materials and methods

Inner Mongolia Agricultural University’s Key Laboratory of Grassland and Resources of the Ministry of Education cultivated wild *G. maritima* seedlings in 1/2 Hoagland solution in a greenhouse for 15 days (wild G. maritima seedlings were collected from the Hailiutu base of Inner Mongolia Agricultural University Science and Technology Park, Bikeqi Town, Tumet Left Banner, Hohhot, Inner Mongolia, N:40° 38 ′, E:111° 28 ′, Height:1060 m.). The optimal growing circumstances included a steady 25°C temperature, 60% relative humidity, and 1600 lx of light. Selected *G. maritima* seedlings were subjected to NaCl concentrations of 0, 200, 400, 600, and 800 mM during 24 hours. Malondialdehyde (MDA), Pro, conductivity, and the activity of superoxide dismutase (SOD), peroxide (POD), glutathione (GSH) reductase (GPX), and catalase (CAT) were measured in *G. maritima* leaf tissues of 0.2 g Each index measurement was repeated thrice for each of the five treatments (0, 200, 400, 600, and 800 mM NaCl).

### Paraffin sections

Fresh leaf tissue of *G. maritima* was fixed with FAA fixative (formaldehyde: glacial acetic acid: ethanol = 0.5:0.5:9) for > 24 h. The blades and labels were placed in the fume hood into the dehydration tank for dehydration and wax soaking. After being dried out, samples were subjected to various chemical treatments, including 4 hours in 75% ethanol, 2 hours in 85% ethanol, 2 hours in 90% ethanol, 1 hour in 95% ethanol, 30 minutes in anhydrous ethanol I, and ethanol II, 5-10 minutes in alcohol benzene, and 5-10 minutes in xylene II. The wax was soaked for an hour in paraffin I, II, and III, all melted at 65° C. After embedding, using a paraffin microtome, thin slices (4 μm) were cut from the samples. Finally, the slices were flattened in 40 °C warm water, dried in a 60 °C oven, and then retrieved and stored at room temperature. Later, an optical microscope was used to examine the cells (Liu, 2006).

### Determination of MDA, proline, and relative conductivity

Fresh plant leaf tissue (0.2 g) was homogenized at 4°C in 5% (w/v) trichloroacetic acid (TCA) (10 mL) to determine malondialdehyde (MDA). Two ml of the supernatant and two ml of 0.67 percent (m/v) thiobarbituric acid (prepared in 10% TCA) were added to the homogenate after centrifugation at 4000 rpm for 10 minutes. The samples were heated to 95°C for 20 minutes before being cooled to 25°C in an ice bath. After re-centrifuging the samples for 5 minutes at 4000 rpm, the absorbance at 450, 532, and 600 nm was measured (Khan et al., 2021). To calculate the MDA concentration, the absorption coefficient was used i.e.,


MDA(μmol/g)=6.45×Δ(A532nm−A600nm)/0.56×A450nm

To calculate proline concentration, we homogenized 0.2 g of fresh plant leaf tissue in 3.0% sulphosalicylic acid (2.5 ml). The homogenate was centrifuged for 10 minutes at 1000 rpm/min. Two ml of the supernatant, two ml of glacial acetic acid, and three ml of acid ninhydrin were placed in a test tube. The mixture was heated for 30 minutes in a 100°C water bath before being cooled rapidly in an ice bath. Initially, the solution was chilled, and afterwards, 4 mL of toluene was poured prior to vortexed. A fresh test tube was used for the toluene (top layer) containing the chromophore. In the end, absorbance at 520 nm was determined using toluene as a standard in a spectrophotometer. As a result of employing a standard curve, the proline content was calculated and reported in mg·g^-1^ (Khan et al., 2021).

20 new leaves were chosen at random, trimmed with scissors, added to 20 ml of deionized water, and let to soak for 24 hours before the conductivity of the resulting solution was recorded as R1. They were given a 10-minute boil-water bath before being allowed to remain at room temperature before another conductivity measurement was taken and the result was recorded as R2. The following equation was used to get the relative conductivity: (Ma et al., 2003)


Relative conductivity=R1/R2×100%

### Determination of SOD, POD, CAT, and APX enzyme activity

Liquid nitrogen was used to crush fresh leaves (0.2 g), which were then dissolved in PBS and centrifuged for 10 minutes at 4°C at 12000 rpm/min. The antioxidant enzymes superoxide dismutase (SOD), catalase (CAT), and ascorbic acid peroxidase (APX) were tested using a nitroblue tetrazolium (NBT) light, dioxygen water, and ascorbic acid reduction method (APX) (McCord and Fridovich, 1969; Beers and Sizer, 1952; Chance and Maehly, 1955; Nakano and Asada, 1981).

### RNA isolation

**Figure 1:**
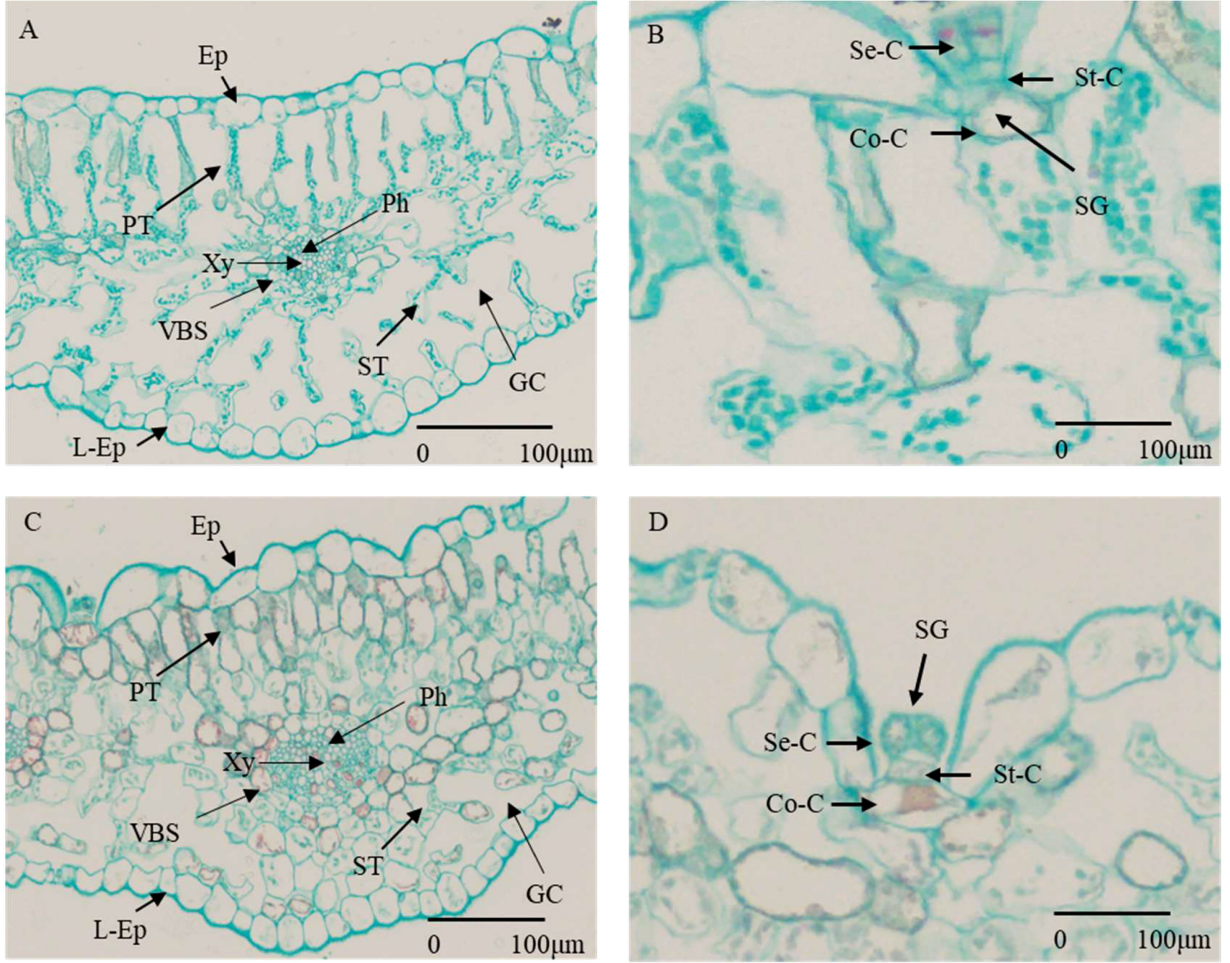
Cross section of *G*. *maritima* leaves under different salt concentrations. **(A)** State of various cells under 0 mM/L NaCl stress. (Ep, epidermis; L-Ep, lower epidermis; PT, palisade tissue; Ph, phloem; Xy, xylem; VBS, vascular bundle sheath; ST, spongy tissue; GC, gas chamber.) **(B)** Composition and status of salt glands under 0 mM/L NaCl stress (SG, salt gland; Se-C, secretory cell; St-C, stalk cell; Co-C, collecting cells.) **(C)** State of various cells under 800 mM/L NaCl stress (abbreviations are the same as for **A**). **(D)** Composition and status of salt glands under 800 mM/L NaCl stress (abbreviations are the same as for **B**).

Total RNA was extracted using a TRIzol reagent kit (Invitrogen, Carlsbad, CA, USA), as per the manufacturer’s instructions (Pandey et al., 2022). To verify the integrity of the RNA, we processed it through an RNase-free agarose gel electrophoresis and then analyzed the results using an Agilent 2100 Bioanalyzer (Agilent Technologies, Palo Alto, CA, USA). Following total RNA isolation, While Oligo(dT) beads were used to remove rRNA from eukaryotic mRNA, the Ribo-ZeroTM Magnetic Kit was used to do the same for bacterial mRNA (Epicenter, Madison, WI, USA) (Attached **Figure 1**, **Supplementary Table 1**). The enriched mRNA was fragmented into tiny bits using fragmentation buffer before being used for reverse transcription into cDNA. Second-strand cDNA was synthesized using DNA polymerase I, deoxynucleoside triphosphates, RNase H, and a buffer. Furthermore, the extraction of cDNA was carried out via QiaQuick PCR extraction kit (Qiagen, Venlo, The Netherlands), then it was end-repaired, had an extra base added, and finally, Illumina sequencing adapters were utilized to ligate it. Finally, Size-selected agarose gel electrophoresis was used to isolate the ligation products, which were then amplified by PCR and sequenced on an Illumina Novaseq 6000. (Gene Denovo Biotechnology, Guangzhou, China).

### Data analysis

Excel 2019 was used for preliminary data processing. Several variables were analyzed using univariate analysis with SPSS (version 19.0; SPSS, Chicago, Illinois, USA) to demonstrate their importance across a range of salt stress treatments, and the differential gene statistics were based on the results of differential analysis. The screening of significantly DEG was based on | log2FC|>1 and FDR < 0.05. The expression patterns of the gene were clustered using trend analysis, followed by analysis using the short time series expression miner tool, then enrichment using KEGG and GO terms. In order to get the enriched GO keywords and pathways, we used a threshold of Q ≤ 0.05.

### qRT−PCR verification

*TransScript* One-Step gDNA Removal and Cdna Synthesis SuperMIX was used to reverse transcribe RNA. *PerfectStart* Green qPCR SuperMIX was used, which contained a 20 μL solution (50 ng cDNA as a template;10 × ROX reference dye), for qRT-PCR in a LightCycler 480 (Roche). This was conducted on each sample. The amplification protocol was as follows: 94°C for 30 sec, then 40 cycles at 94°C for 5sec, 60°C for 34sec and 72°C for 10 s, and then melt curve at 94°C 15 s, 60°C 1 min, and 94°C 15 s. **Supplementary Table 4** lists the sequence of the specific primers used for qRT-PCR. β-Actin was used as an internal control. All qRT-PCR analyses were performed in three replicates. The relative gene expression levels were calculated using the 2^−ΔΔCt^ method.

## Results

### Plant morphological changes under salt stress

**Figure 2:**
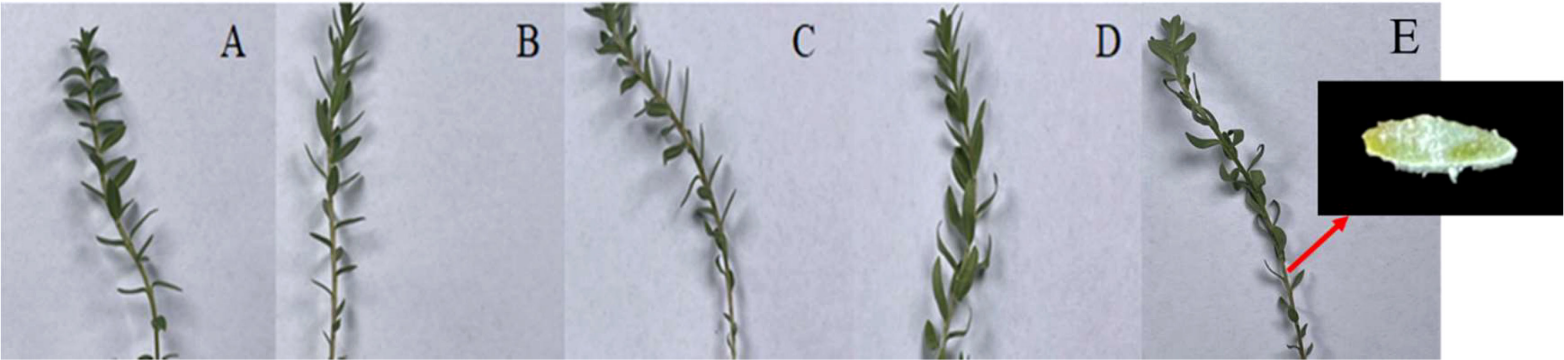
Changes in *G. maritima* leaves after 24 h under salt stress. **(A–E)** Leaf morphology of *G. maritima* under 0, 200, 400, 600, and 800 mM/L NaCl stress. The small picture in the lower left corner shows the salt secretion state of a single leaf.

The observation of changes in plant phenotype under abiotic stress was an important indicator for evaluating plant tolerance. By observing the transverse section of the plant leaf tissue, we can better observe the salt-tolerant structure of halophytes (Zhao, 2016). As a salt-secreting halophyte, with the increase of salt stress concentration, the phenotype of *G. maritima* seedlings were changed (**Figures 2A–E**). The leaves of *G. maritima* seedlings curled and salted out under 800 mM NaCl stress (**Figure 2E**). When observing the cell structure of the leaves of *G. maritima* (**Figures 1A–D**), it was found that the salt glands were the main salt-excreting tissue. The tissue was trapped in EC, which were composed of stalk cells (ST-Cs), collecting cells (Co-Cs), and secretory cells (SE-Cs) (**Figure 1B, D**).

**Figure 3:**
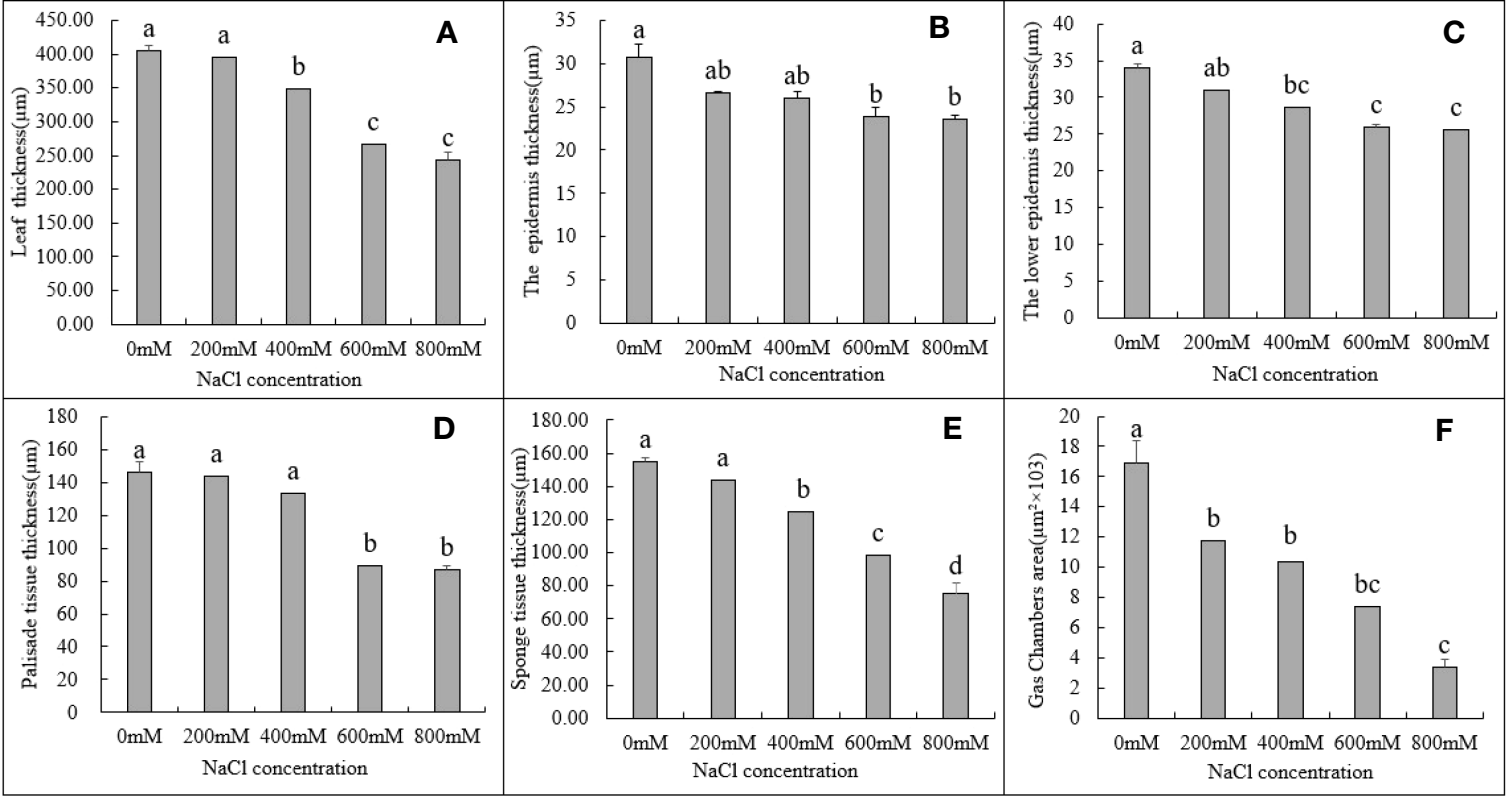
Changes in various tissues in the leaves of *G. maritima* under different salt concentrations stress. **(A)** The thickness in the leaves of *G. maritima* under different salt concentrations stress; **(B, C)** The epidermis and lower epidermis in the leaves of *G. maritima* under different salt concentrations stress; **(D)** Palisade tissue in the leaves of *G. maritima* under different salt concentrations stress; **(E)** Sponge tissue in the leaves of *G. maritima* under different salt concentrations stress; **(F)** Gas chamber area in the leaves of *G. maritima* under different salt concentrations stress. Different letters indicate significant differences under different salt concentrations (*P < 0.05*).

The thickness of leaf, the epidermis and lower epidermis, palisade tissue, and spongy tissue, as well as the size of the air cavity, all decreased in *G. maritima* leaves as the salt concentration increased (**Figure 3A–D, F**). The thickness of the upper and lower EC, as well as the size of the air cavity, were substantially decreased by 13.31%, 8.93%, and 30.81% and 14.94%, 15.68%, and 38.61%, after being treated to 200 and 400 mM NaCl, respectively, compared to control (**Figure 3B, C, F**, P < 0.05). Under treatment with 600 and 800 mM NaCl, the leaf thickness decreased by 33.90% and 39.76%, respectively, compared to the control. Upper EC thickness was decreased by 22.06% and 23.33% (*P* < 0.05) with reference to the control group (**Figure 3B**), the thickness of the lower EC decreased significantly by 23.99% and 24.64% (**Figure 3C**), the thickness of the palisade tissue decreased significantly by 38.63% and 40.74% (**Figure 3D**), and the thickness of the spongy tissue decreased by 36.36% and 51.50%, respectively (**Figure 3E**). Compared to the control treatment, the cavity area decreased the most (56.20% and 80.28%, respectively) (**Figure 3F**).

### Physiological changes under salt stress

**Figure 4:**
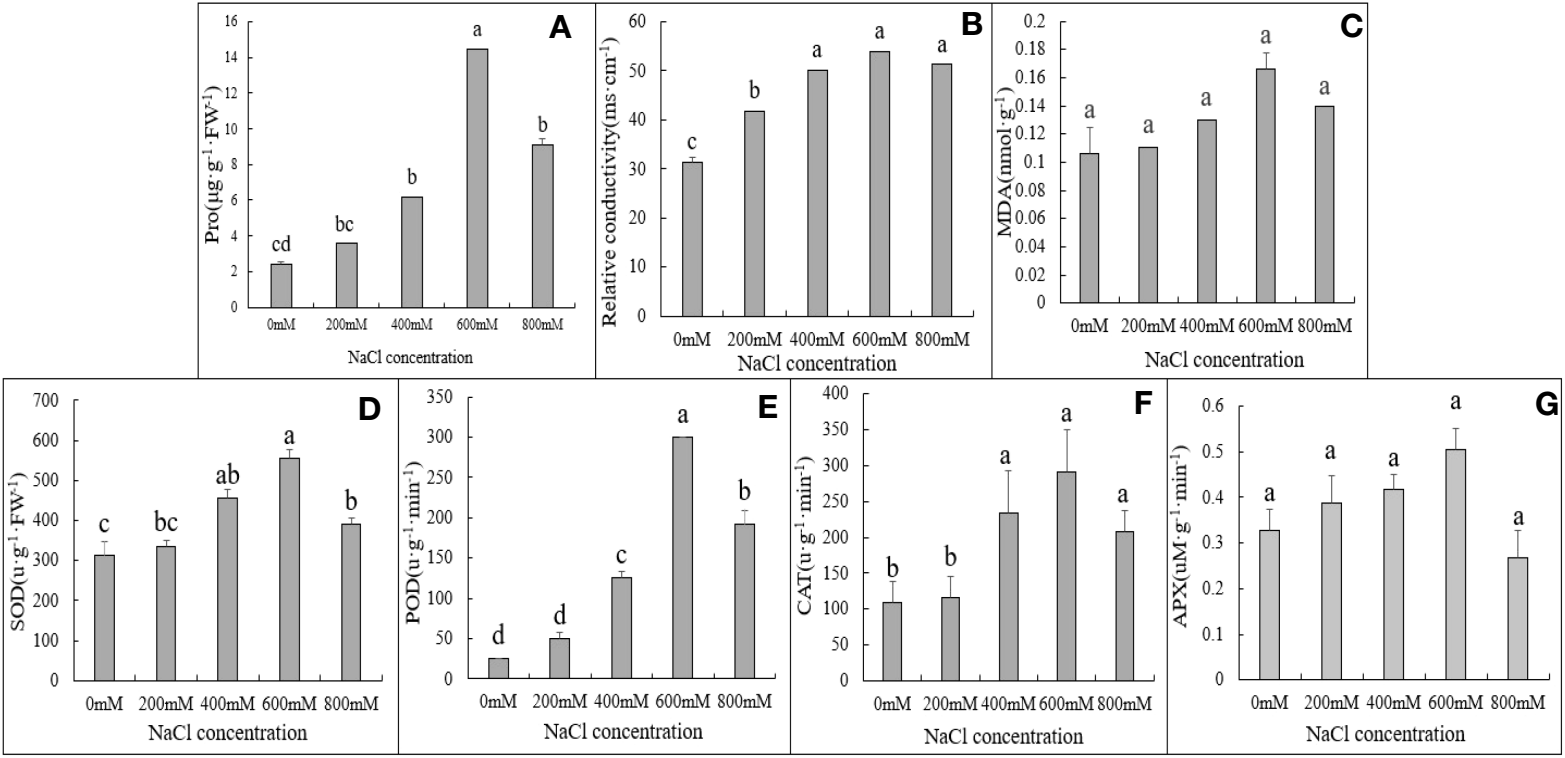
Physiological and biochemical indexes of *G*. *maritima* leaves under salt stress. **(A)** proline content of *G*. *maritima* leaves under salt stress; **(B)** Relative conductivity of *G*. *maritima* leaves under salt stress; **(C)** MDA of *G*. *maritima* leaves under salt stress; **(D)** SOD enzyme activity of *G*. *maritima* leaves under salt stress; **(E)** POD enzyme activity of *G*. *maritima* leaves under salt stress; **(F)** CAT enzyme activity of *G*. *maritima* leaves under salt stress; **(G)** APX enzyme activity of *G*. *maritima* leaves under salt stress. Different letters indicate significant differences under different salt concentrations (*P < 0.05*).

In addition to the phenotypic changes observed in plants under salt stress, ROS, malondialdehyde, and cell membranes in plants initiate corresponding tolerance mechanisms (Gong et al., 2014; Zou et al., 2020). Physiological indicators such as proline and malondialdehyde contents and plant antioxidant enzyme activity may be used to evaluate the salt tolerance mechanisms of *G. maritima*. Proline concentration was highest under 600 mM NaCl stress. Up to 600 mM NaCl, activity was significantly higher, however, they were lower at 800 mM NaCl, and proline content significantly increased in the leaves of *G. maritima* in all NaCl treatments in comparison to the control group (*p* < 0.05; **Figure 4A**, **Table 1**).

**Table 1:** Physiological and biochemical indexes of *G. maritima* leaves under salt stress.

	0mM	200mM	400mM	600mM	800mM
MDA (nmol·g^-1^)	0.11 ± 0.018a	0.12 ± 0.012a	0.13 ± 0.004a	0.17 ± 0.013a	0.14 ± 0.015a
Pro(μg·g^-1^·FW^-1^)	2.43 ± 0.13cd	3.56 ± 0.30bc	6.15 ± 0.81b	14.47 ± 0.61a	9.11 ± 0.86b
Relative conductivity(ms·cm^-1^)	31.25 ± 1.21c	41.81 ± 0.77b	50.01 ± 0.61a	54.01 ± 0.71a	51.44 ± 0.53a
SOD(μ·g^-1^·FW^-1^)	312.99 ± 33.01c	336.16 ± 14.76bc	456.78 ± 20.56ab	554.80 ± 23.35a	390.11 ± 16.99b
POD(μ·g^-1^·min^-1^)	25 ± 1.45d	50.00 ± 8.33d	125 ± 8.33c	300 ± 4.44a	191.67 ± 17.35b
APX (μM·g^-1^·min^-1^)	0.33 ± 0.05a	0.39 ± 0.06a	0.42 ± 0.03a	0.51 ± 0.05a	0.27 ± 0.06a
CAT(μ·g^-1^·min^-1^)	108.33 ± 29.27b	116.67 ± 29.27b	233.33 ± 59.12a	291.67 ± 58.53a	208.33 ± 29.67a

Different letters indicate significant differences between treatments (P < 0.05).

Relative conductivity analysis revealed that salt stress substantially increased lipid peroxidation in *G. maritima* leaves (*p < 0.05*). In the leaves of *G. maritima*, relative conductivity rose up to 600 mM NaCl in comparison to the control and remained noticeably high under all salt treatments (*p < 0.05*; **Figure 4B**, **Table 1**). The MDA concentration of *G. maritima* did not change between the salt stress treatments and the control, however (*p> 0.05*; **Figure 4C**, **Table 1**). Under various NaCl treatments, a similar trend was seen for the POD and SOD activity. Up to 600 mM NaCl, SOD and POD activity were significantly higher than control (*p < 0.05*), however they were lower at 800 mM NaCl. To a concentration of 600 mM NaCl, interestingly, POD activity was 12 times greater than the control and SOD activity was 1.8 times higher (**Figure 4D, E**, **Table 1**).

Under high salt concentration stress (400-800mM NaCl), the CAT enzyme activity of *G. maritima* seedlings was significantly higher than that of the control (*p<0.05*). And at 600 mM NaCl, CAT activity reached its maximum, which increased by 1.69 times compared to the control (**Figure 4F**, **Table 1**). As the salt concentration increased, there was no significant difference in APX activity among different treatments(*p*>0.05) (**Figure 4G**, **Table 1**).

### Transcriptome sequencing in *G. maritima* under salt stress

Based on the proline content and SOD and POD activities after different treatments, fresh *G. maritima* leaves after 24 h of 600 and 800mM NaCl treatments were collected; 0mM NaCl was used as the control. The RNA-seq experiment included nine libraries, one for each of the three replicates of each sample (N0: control; N3: plants under 600mM NaCl stress; and N4: plants under 800 mM NaCl stress). Subsequently, Total raw data sequences ranged from 36,348,544 to 49,426,720, yielding 6.23–8.48 GB of sequencing data (**Supplementary Table 2**). All the cDNA libraries yielded a total of 176,208 transcripts. After ensuring their integrity, the transcripts were combined into 79,986 unigenes with a N50 of 1,732 bp. **Table 2** provides a brief overview of the transcriptome sequencing of *G. maritima* (**Table 2**).

**Table 2:** Characteristics of *G. maritima* transcriptome sequencing.

Index	Transcipts	Gene
All	176,208	79,986
GC%	38.57	38.22
Max length (bp)	16,681	16,681
Median length (bp)	667	561
Min length (bp)	183	201
Total assembled bases	220,150,171	76,483,799
N50 (bp)	2,078	1,732

All assembled unigene clusters were aligned using BLASTX with an E-value cutoff of 0.00001 against orthologous group clusters for eukaryotic complete genomes SwissProt database (http://www.expasy.ch/sprot/), KEGG (http://www.genome.jp/kegg/), (GOG/KOG, (http://www.geneontology.org), Pfam database (http://pfam.xfam.org/), evolutionary genealogy of genes, and NCBI non-redundant protein database (NCBI_NR, http://www.ncbi.nlm.nih.gov/). The statistical findings from the six reputable databases are shown in **Table 3**.

**Table 3:** Statistical results from gene annotation.

	Number	Ratio%
All	35,087	100
COG/KOG	20,310	25.39
KEGG	33,874	42.35
Pfam	34,681	43.36
SwissProt	23,768	29.72
NR	34,591	43.25

### Differentially expressed genes in *G. maritima* under salt stress

**Figure 5:**
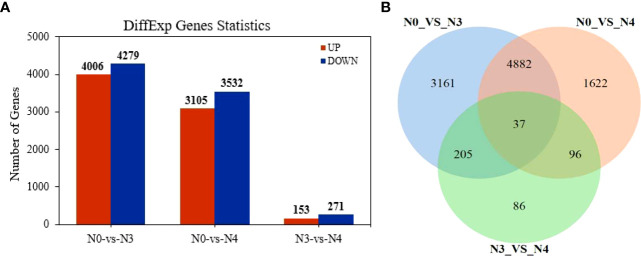
Differentially expressed genes in transcriptome data of *G*. *maritima* leaves under salt stress. **(A)** Statistics of differential genes after comparison at three concentrations; **(B)** Venn diagram of differential genes after comparison at three concentrations. N0:0 mM/L NaCl stress; N3:600mM/L NaCl stress; N4:800mM/L NaCl stress.

Differentially regulated genes in *G. maritima* in response to salt stress were found by comparing three sets of experimental conditions (N0, N3, and N4). Ten thousand eight hundred and nine genes’ expressions were found to be substantially varied in the three groups. Specifically, the comparisons between N0 and N3, N0 and N4, and N3 and N4 yielded 8285, 6637, and 402 DEGs, respectively (**Figure 5A**). **Figure 5B** presents a Venn diagram showing the overlap between the DEGs from the three comparisons. Between N0 and N4, there were 4,919 DEGs of overlap. Thirty-seven differentially expressed genes (DEGs) were found to be affected by therapy (**Figure 5B**). **Table 4** displays data pertaining to the 37 DEGs throughout the three analyses (**Table 4**).

**Table 4:** Comparison of the N0 vs. N3, N0 vs. N4, and N3 vs. N4 groups regarding common differential gene ID, differential multiple, and participating pathways.

Gene ID	N0	N3	N4	Symbol	Pathway
Unigene0007175	0	-5	-3.07	*ABP19A*	–
Unigene0009759	0	-7.65	-2.82	*HPR3*	Glycine, serine, and threonine metabolism, Carbon metabolism, Metabolic pathways; Biosynthesis of secondary metabolites; Glyoxylate and dicarboxylate metabolism
Unigene0010874	0	4.69	8.34	*–*	–
Unigene0011428	0	3.94	5.93	*GT6*	–
Unigene0013930	0	-4.11	-2.06	*At4g24780*	Pentose and glucuronate interconversions Metabolic pathways
Unigene0015412	0	4.98	3.42	*LTP3*	–
Unigene0018706	0	-1.14	-6.88	*–*	–
Unigene0019049	0	1.96	3.7	*PIP1-1*	–
Unigene0019050	0	2.39	4.21	*PIP1.4*	–
Unigene0022950	0	-3.54	-1.73	*SPS2*	Biosynthesis of secondary metabolites; Starch and sucrose metabolism, Metabolic pathways;
Unigene0023204	0	1.87	-1.78	*–*	–
Unigene0023422	0	7.73	3.87	*LCR71*	–
Unigene0024987	0	1.03	2.15	*–*	–
Unigene0025676	0	2.98	1.93	*At1g64890*	–
Unigene0026149	0	-5.57	-2.99	*At4g00165*	–
Unigene0028635	0	-2.4	-1.38	*ICR2*	–
Unigene0033159	0	2.89	4.25	*–*	–
Unigene0033609	0	5.75	4.55	*–*	–
Unigene0034631	0	6.59	7.88	*XTH24*	Plant hormone signal transduction
Unigene0034632	0	4.56	6.68	*XTH25*	Plant hormone signal transduction
Unigene0034645	0	3.53	6.1	*THE1*	–
Unigene0039722	0	3.36	2.19	*NPF6.2*	–
Unigene0040492	0	3.51	1.52	*–*	–
Unigene0044801	0	6.72	4.14	*HIPP23*	–
Unigene0046393	0	1.09	2.59	*ERD7*	Endocytosis
Unigene0047762	0	-2.55	2.7	*At3g13560*	–
Unigene0049288	0	4.29	7.15	*–*	–
Unigene0057382	0	5.04	8.11	*–*	–
Unigene0061056	0	3.13	5.03	*At4g39010*	Starch and sucrose metabolism Metabolic pathways;
Unigene0061874	0	3.07	4.14	*BCAT2*	Biosynthesis of secondary metabolites; Metabolic pathways; Cysteine and methionine metabolism; Valine, leucine and isoleucine biosynthesis; Biosynthesis of amino acids; leucine and isoleucine degradation; Pantothenate and CoA biosynthesis; 2-Oxocarboxylic acid metabolism; Glucosinolate biosynthesis
Unigene0069678	0	4.68	2.88	*PMEI11*	–
Unigene0071107	0	8.6	6.67	*–*	–
Unigene0073221	0	2.6	1.18	*LTP2*	–
Unigene0074776	0	3.21	4.97	*–*	–
Unigene0075998	0	7.21	4.84	*–*	–
Unigene0078325	0	5.27	3.77	*UGE2*	Metabolic pathways; Amino sugar and nucleotide sugar metabolism; Galactose metabolism
Unigene0079898	0	-2.39	-4.14	*SOT15*	–

N0:0 mM/L NaCl stress; N3:600mM/L NaCl stress; N4:800mM/L NaCl stress.

### Gene expression under different salt stress treatments

**Figure 6:**
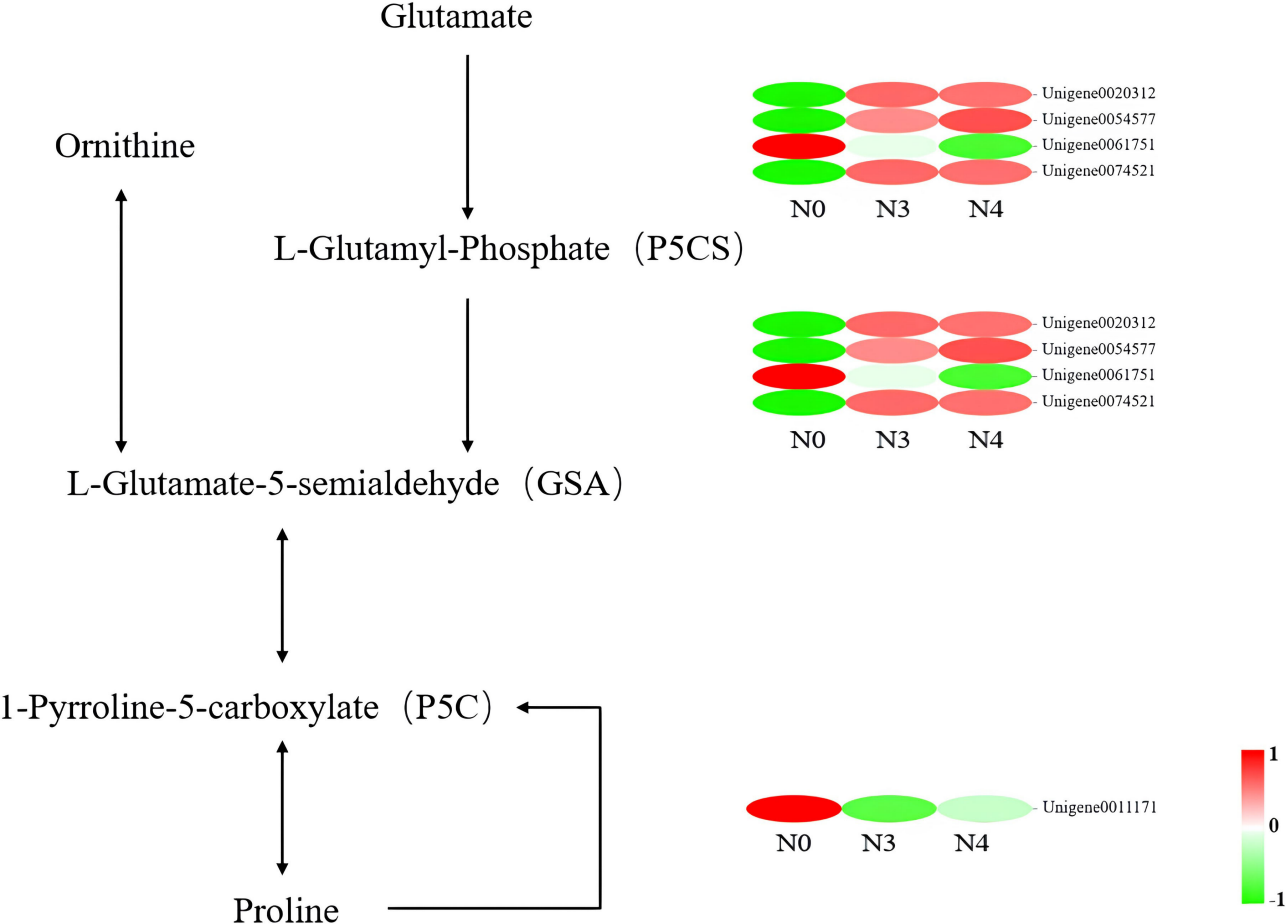
Heat map of differential gene expression in the arginine and proline metabolic pathways. N0:0 mM/L NaCl stress; N3:600mM/L NaCl stress; N4:800mM/L NaCl stress.

“Arginine and proline metabolism” and “peroxisome” were the primary key pathways identified. In the “arginine and proline metabolism” pathways, L-glutamate-5-semialdehyde encoding genes (*δ-oat*, Unigene0003065; *PRO*, Unigene0020312, Unigene005477, and Unigene0074521) all saw increases in their expression levels. One gene encoding L-glutamate-5-semialdehyde and L-glutamyl-phosphate also had its expression reduced in response to 800 mM NaCl stress (*PRO2*, Unigene0061751). Another downregulated gene encodes enhanced 1-pyrroline-5-carboxylate, which is involved in proline biosynthesis (*POX2*, Unigene0011171) (**Figure 6**, **Table 5**).

**Table 5:** Differential multiples and pathways of differential genes affecting proline synthesis.

Gene ID	N0	N3	N4	Symbol	Pathway
Unigene0003065	0	2.13	1.91	*δ-oat*	Metabolic pathways; Biosynthesis of secondary metabolites Arginine and proline metabolism
Unigene0011171	0	-2.66	-1.32	*POX2*	Metabolic pathways; Biosynthesis of secondary metabolites; Biosynthesis of amino acids; Arginine and proline metabolism
Unigene0020312	0	4.24	4.21	*PRO2*	Metabolic pathways; Biosynthesis of secondary metabolites; Biosynthesis of amino acids; Arginine and proline metabolism
Unigene0054577	0	3.07	3.26	*PRO2*	Metabolic pathways; Biosynthesis of secondary metabolites; Biosynthesis of amino acids; Arginine and proline metabolism
Unigene0061751	0	-0.52	-1.03	*PRO2*	Metabolic pathways; Biosynthesis of secondary metabolites; Arginine and proline metabolism
Unigene0074521	0	4.09	4.06	*PRO2*	Metabolic pathways; Biosynthesis of secondary metabolites; Biosynthesis of amino acids; Arginine and proline metabolism

N0:0 mM/L NaCl stress; N3:600mM/L NaCl stress; N4:800mM/L NaCl stress.

Genes related to peroxisomes under salt stress conditions were further investigated. In the “peroxisome” pathway, five genes affected SOD and CAT activities. These genes mainly affected the PTS1 type hydrogen peroxide metabolism in the antioxidant system of peroxisomal proteins. The expression levels of two genes encoding CAT were upregulated. These genes also affected H_2_O_2_ synthesis in the “MAPK signaling pathway – plant” pathway. Three negatively regulated genes encode SOD (*MSD1*, Unigene0034918) and *SODCC.2* (Unigene0017655, Unigene0031302). Except for *SODCC.2* (Unigene0017655), which was downregulated, the other two genes were upregulated (**Table 6**).

**Table 6:** Differential multiples and pathways of differential genes in the peroxisome pathway.

Gene ID	N0	N3	N4	Symbol	Pathway
Unigene0002691	0	1.75	2.49	*CAT1*	Glyoxylate and dicarboxylate metabolism; Biosynthesis of secondary metabolites; Metabolic pathways; Carbon metabolism; MAPK signaling pathway - plant; Peroxisome; Tryptophan metabolism
Unigene0057791	0	1.17	1.37	*CAT3*	Carbon metabolism; MAPK signaling pathway - plant; Glyoxylate and dicarboxylate metabolism; Metabolic pathways; Biosynthesis of secondary metabolites; Peroxisome; Tryptophan metabolism
Unigene0017655	0	-1.35	-0.88	*SODCC.2*	Peroxisome
Unigene0031302	0	2.38	1.72	*SODCC.2*	Peroxisome
Unigene0034918	0	1.21	2.94	*MSD1*	Peroxisome

N0:0 mM/L NaCl stress; N3:600mM/L NaCl stress; N4:800mM/L NaCl stress.

### Trends in DEGs under different levels of salt stress

**Figure 7:**
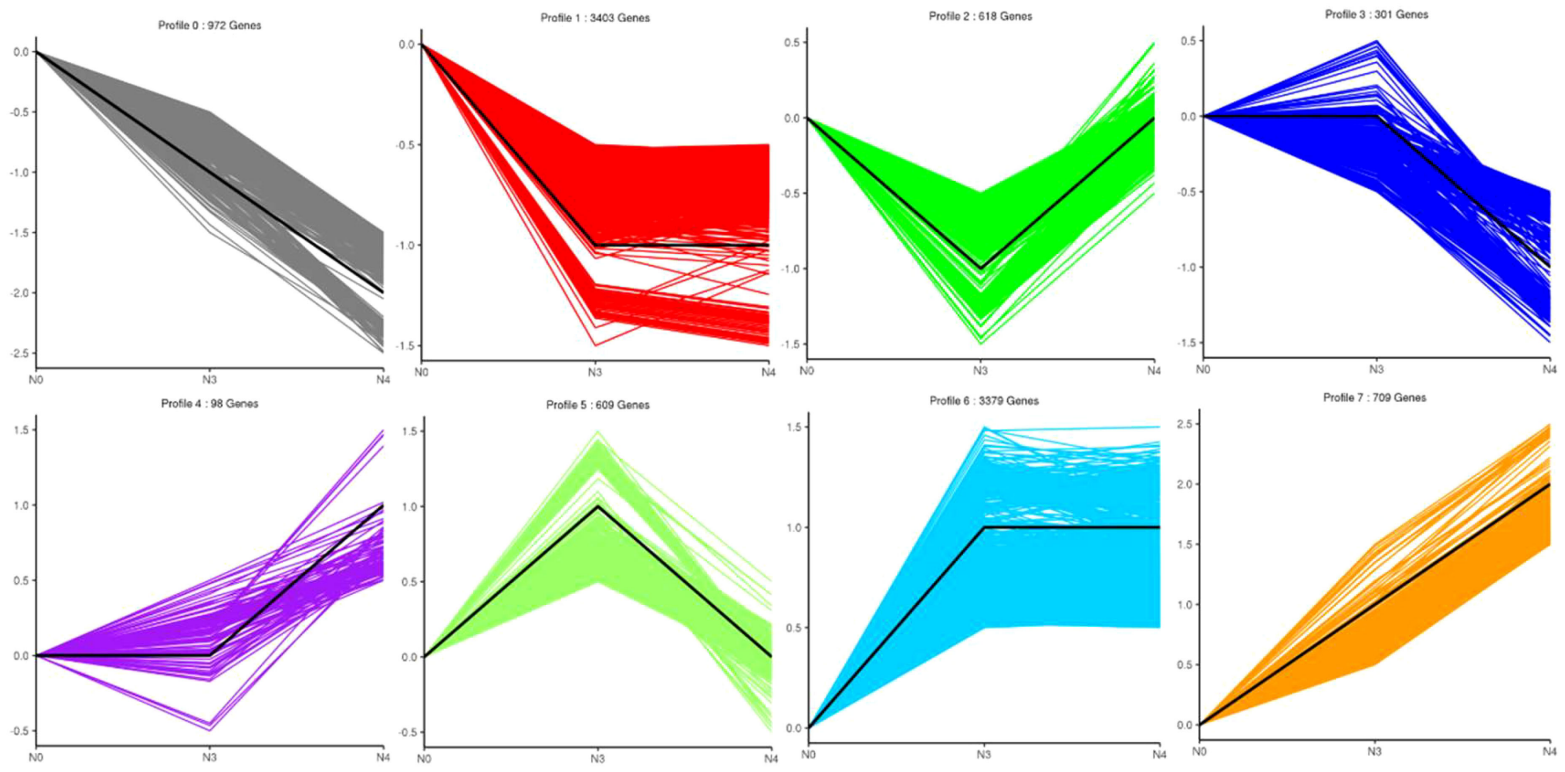
Number and trend of genes counted after trend analysis of transcriptome data of *G. maritima* after salt stress. N0:0 mM/L NaCl stress; N3:600mM/L NaCl stress; N4:800mM/L NaCl stress.

**Figure 8:**
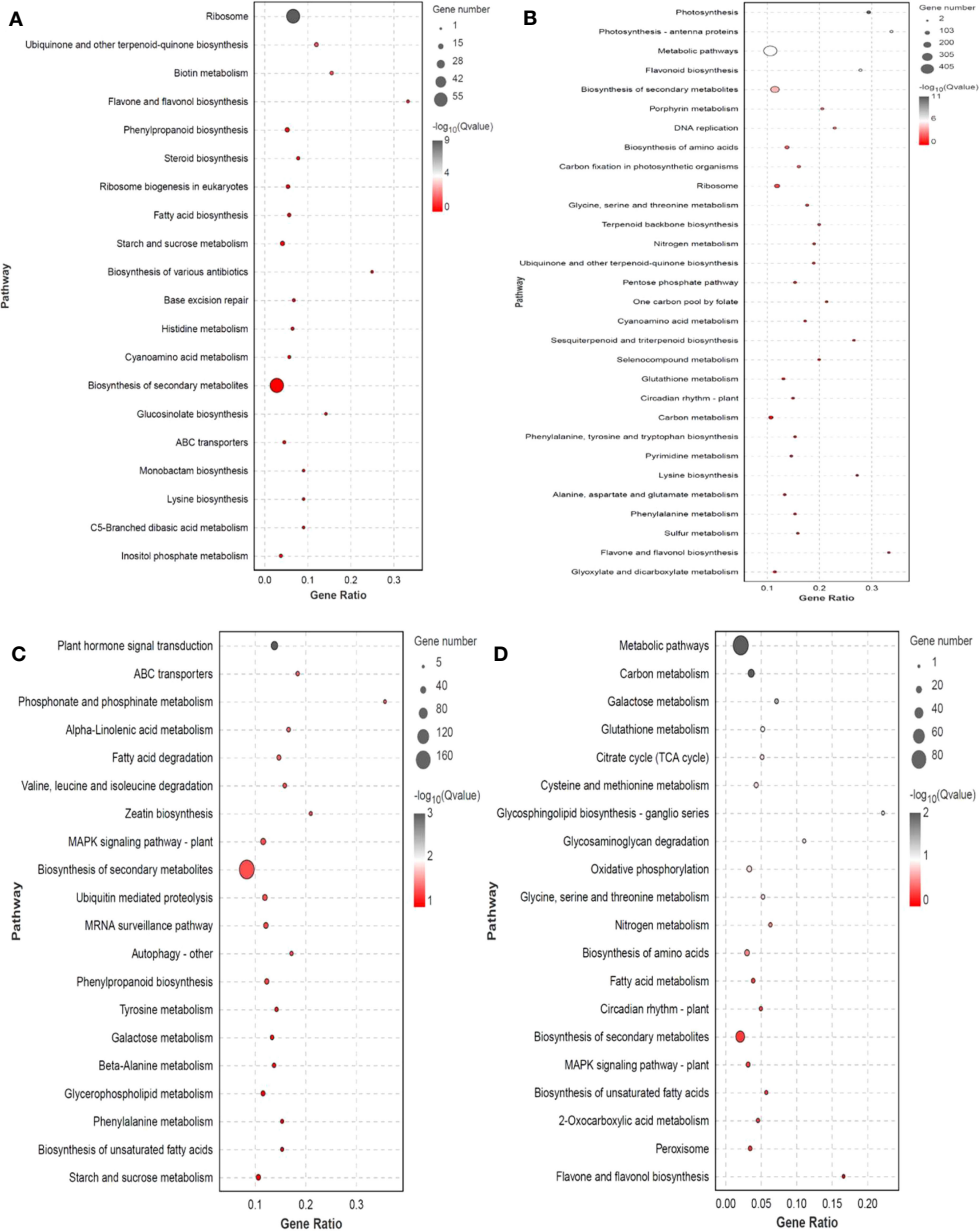
KEGG enrichment significance bubble chart of four trends (profiles 0, 1, 6, and 7) with significant differences after trend analysis. **(A)** profile 0 KEGG enrichment significance bubble charts; **(B)** profile 1 KEGG enrichment significance bubble charts; **(C)** profile 6 KEGG enrichment significance bubble charts; **(D)** profile 7 KEGG enrichment significance bubble charts.

DEG expression patterns were identified using trend analysis by comparing DEGs at 24 h under varying NaCl concentrations. Most DEGs were enriched in profiles 0, 1, 6, and 7 when G. maritima was exposed to salt stress (*P< 0.05*). (**Figure 7**). KEGG analysis showed a distinct upward trend in expression (**Figure 8**), with profiles 7 and 6 showing considerable enrichment of DEGs. Critical pathways involved in disease resistance were also significantly enhanced, including “MAPK signaling pathway-plant” (37 DEGs), “glutathione metabolism” (8 DEGs), and “plant hormone signal transduction” (48 DEGs) (**Figures 8C, D**). Similar attention was paid to profile 0 since DEGs enriched there showed a distinct downregulated expression pattern (**Figure 7**). In addition, 4 profiles indicated enrichment for “flavone and flavanol biosynthesis” (2 DEGs) and “flavonoid biosynthesis” (24 DEGs), whose metabolites affect abiotic stress response (**Figures 8A, B**).

**Figure 9:**
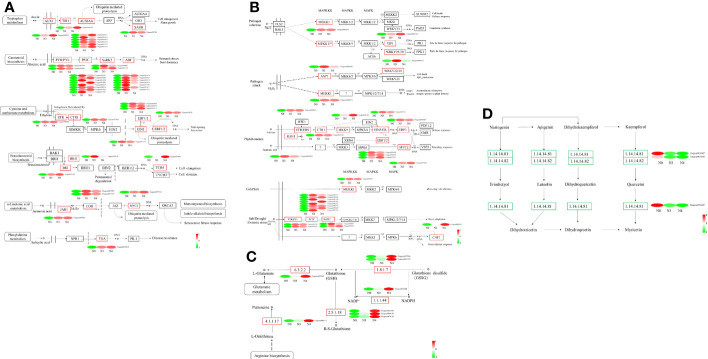
Heat map of differential gene expression in “the Plant hormone signal pathway”, “MAPK signaling pathway-plants”, “Glutathione metabolism pathway” and “flavone and flavonol biosynthesis pathway”. **(A)** the Plant hormone signal pathway, **(B)** MAPK signaling pathway-plants, **(C)** Glutathione metabolism pathway, **(D)** flavone and flavonol biosynthesis pathway. N0:0 mM/L NaCl stress; N3:600mM/L NaCl stress; N4:800mM/L NaCl stress.

Key mechanisms found under salt stress include the “glutathione metabolism,” and “plant hormone signal and MAPK signaling pathway - plant,”. The 14 genes encoding mitogen-activated protein kinases (MAPK), three genes encoding inhibitors of ethylene synthesis, one gene that indirectly affects the inhibition of phosphorylation in jasmonic acid synthesis (*MYC2*), 15 genes inhibiting dephosphorylation, and one gene indirectly inhibiting H_2_O_2_ synthesis (*CAT1*) in ABA synthesis under abiotic stress were upregulated. The “plant hormone signal transduction” pathway had 48 genes that were substantially enriched in *Arabidopsis* (*P<0.05*), four of which are involved in abscisic acid and ethylene, including those encoding protein phosphatase (PP2C), protein kinase (SnRK2), and ethylene receptors (ETR). Enzymes for the production and breakdown of auxin, cytokinin, brassinosteroid, jasmonic acid, and salicylic acid are encoded by other genes. A total of three genes are involved in GSSG reactions along the “glutathione metabolism” pathway, the three genes are related to GSH, one gene is involved in arginine biosynthesis, and one gene participates in glutamate metabolism. These genes were also upregulated (**Supplementary Table 3**; **Figures 9A–C**). In profile 0, DEGs were considerably enriched (P<0.05). Flavone and flavonol biosynthesis, as well as flavonoid biosynthesis, the expression levels of 25 DEGs encoding *CHS, CHS1, CHS2, ANR, DRF, LAR, LDOX, F3H-2, CCOMT*, and *SALAT* were downregulated (**Supplementary Table 4**; **Figure 9D**).

### RT-qPCR validates RNA-seq data

**Figure 10:**
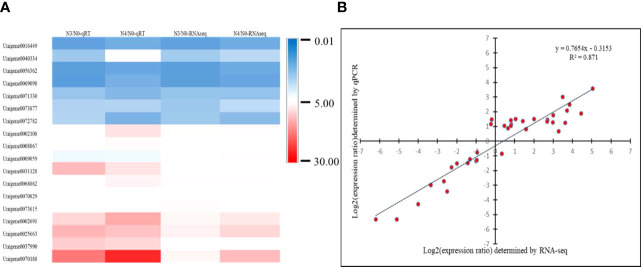
shows a heatmap of unigene expression to verify the accuracy of RT-qPCR. **(A)** Heatmap of 18 differently expressed unigenes’ expression levels; **(B)** correlation of 18 differentially expressed unigenes as determined by RNA sequencing and quantitative RT-qPCR. N0:0mM/L NaCl stress; N3:600mM/L NaCl stress; N4:800mM/L NaCl stress.

18 DEGs with diverse expression patterns were chosen for RT-qPCR to confirm the RNA-seq data (**Supplementary Table 5**). All of these potential genes were screened from N3 and N4, and qRT-PCR findings and RNA-seq data showed a good positive correlation (R2 = 0.871), indicating the RNA-seq data were repeatable and reliable (**Figure 10**).

## Discussion

### Salt tolerance mechanisms of *G. maritima*

To adapt to an environment with high salt concentrations, some halophytes evolved specific salt-secreting structures (salt glands and salt vesicles). These salt-secreting structures secrete and store Na^+^ to protect the organisms from excessive Na^+^ toxicity (He et al., 2019). After crosscutting the leaves of *G. maritima* seedlings, salt gland tissue that secretes NaCl under salt stress was identified. However, the salt secretion mechanism of salt glands needs to be further explored. In the current work, we more thoroughly examined *G. maritima’s* physiological reaction to salt stress and predicted DEGs that are crucial for this organism’s ability to tolerate salt.

Research on the physiology of halophytes shows that the relative conductivity and proline content, which affect the stability of the plant membrane system, are important physiological indices for evaluating plant salt tolerance. Some studies have shown that changes in relative conductivity reflect the degree of damage to cell membranes under osmotic stress. The relative conductivities of *Vaccinum corymbosum* L., *Graptoveria titubans*, *Ilex chinensis* Sims., *Xanthoceras sorbifolia* Bunge., *Miscanthus sinensis* Anderss., and *Triarrhena sacchariflora* (Maxim.) Nakai leaves after salt stress (Wu, 2019; Zhang, 2019; Xie et al., 2020) have been measured, and the relative conductivity was found to tends to increase with increasing salt concentration, which is consistent with the measurement results of the relative conductivity of *G. maritima* leaves in the present study. This suggests that during salt stress, plant mesophyll cells amass a considerable amount of ions to maintain intracellular equilibrium and limit damage to the plant’s membranes. Under adverse conditions, plants accumulate excessive proline. Proline acts as an osmotic regulator to buffer salt, drought, and other stresses, or as a protector of cytoplasmic enzymes and membranes to minimize damage under adverse conditions. For example, with increasing NaCl concentration, the proline content in *Pugionium cornutum* (L.) Gaertn. leaves increases significantly (Yue et al., 2016). *Rosmarinus officinalis* leaf proline content increases dramatically with increasing stress concentration and time under NaCl (Chetouani et al., 2019). The proline content in *Actinidia chinensis* leaves show an upward trend compared to that in the control under high salt stress (Abid et al., 2020). We demonstrate here that *G. maritima*, when subjected to salt stress, has disruptions in its physiological and metabolic activity. The plant responded by changing its metabolite content. Proline accumulation was one such physiological response. When exposed to a stress concentration of more than 600 mM NaCl, however, the cellular balance of osmotic control and ion regulation was disrupted. the metabolism in cells was disordered, and the proline content was reduced, indicating that the stability of enzymes and the membranes of plant cells may have been lost, and the plant itself could not grow normally at this concentration.

Plants boost antioxidant enzyme activity, eliminate excess ROS, and keep the plant’s internal dynamic equilibrium stable while under stress to prevent cell membrane damage. In order to get rid of unwanted ROS and adapt to adversity, plants rely on antioxidant enzyme systems including SOD, POD, and CAT. Some studies have shown that, as the NaCl stress concentration increases, SOD activity in plant leaves first increases and then decreases. Evidence like this demonstrates that plants under salt stress first respond by keeping their SOD activity high in order to prevent harm; however, high concentrations (800 mM) of salt stress produce O_2_^-^ exceeding the capacity of plants to compensate, dynamic balance is destroyed, and SOD activity is reduced (Zhu et al., 2021). This is consistent with the observed changes in SOD activity in the leaves of *G. maritima* in the present study. As an antioxidant enzyme that further scavenges the antioxidant products of SOD in cells, POD activity first increases and then decreases with increasing salt concentrations. High POD activity was maintained in the leaves of G. maritima seedlings exposed to salt stress, and H_2_O_2_ was cleared in time to prevent more significant OH- toxicity. The normal development of *G. maritima* seedlings was disrupted by exposure to high quantities because of a reduction in POD activity in the leaves, a loss in the plants’ capacity to withstand ROS. The same has also been reported for POD activity changes in plants, such as *Dalbergia granadillo, Poa pratensis* L., and *Hibiscus syriacus* L. under salt damage (Zhao et al., 2017; Lai et al., 2020). CAT is an enzyme scavenger that can remove H_2_O_2_ in plants, promote its decomposition into molecular oxygen and H_2_O, and reduce the toxic effects of H_2_O_2_ on plant cells. Under salt stress, CAT activity in the leaves of *Suaeda glauca* (Bunge) Bunge.*, Matthiolaincana* L. R.Br.*, Hibiscus syriacus* L., and *Mentha haplocalyx* B. first increases and then decreases, which is generally consistent with our results (Jafari and Hashemi Garmdareh, 2019, Pang et al., 2005, Haddadi et al., 2016; Li et al., 2020). This illustrates that when salt concentration grows, the antioxidant system initially acts properly to promote CAT activity, and later, under high salt stress, it can no longer operate normally due to increased H_2_O_2_, resulting in decreased CAT activity, which is similar to the findings of *Hibiscus syriacus* L. (Lu, 2021).

For this research, we used high-throughput transcriptome sequencing to investigate the processes through which *G. maritima* is able to withstand high concentrations of salt. Genes possibly involved in salt stress were identified using the anatomy, physiology, and biochemistry of *G. maritima* leaves as a reference (Zhao et al., 2005). Through the analysis of physiological and biochemical indexes, proline content and SOD and CAT activities in the leaves of *G. maritima* under salt stress were found to be significantly different from those in the control. Transcriptome analysis shows that there were 11 genes affecting proline synthesis, SOD, and CAT activity in the “arginine and proline metabolism” pathway and “peroxisome” pathway, among which six genes affected proline content and regulated SOD and CAT activity. *POX2* expression is consistent with earlier studies indicating that under stress, plants upregulate genes involved in proline synthesis while downregulating those involved in degradation. This gene mainly negatively regulates the activity of proline dehydrogenase (PRODH) to inhibit the degradation of proline to P5C and maintain a high level of proline content to resist the damage caused by salt. In the process of proline production by glutamic acid, the four genes regulating P5CS and GSA were closely related to increased proline content in *G. maritima* after salt stress. Three *PRO2* genes were upregulated, and one gene was downregulated. P5CS regulatory genes that affect changes in proline content (such as *AtP5CS1, OsP5CS, MsP5CS-1, MsP5CS-2, OmPro1, TomPro2, SsP5CS, MtP5CS1, MtP5CS2, PvP5CS1*, and *PvP5CS2*) have also been found in *Arabidopsis thaliana, Oryza sativa* L.*, Lycopersicon esculentum, Suaeda salsa* (L.) Pall.*, diploid alfalfa*, and *Phaseolus vulgaris* L. After salt stress, gene expression and proline content in plants are significantly increased compared to those in the control. In the ornithine pathway, salt stress promotes δ-Oat gene expression to increase the conversion of ornithine aminotransferase to GSA, similar to the findings of Nancy (Roosens et al., 1998). A study of δ-ornithine aminotransferase in *Arabidopsis* under salt stress show similar results. In the present study, the five genes affecting the antioxidant enzyme system included two upregulated catalases (*CAT1* and *CAT3*), two upregulated superoxide dismutases (*SOD1* (Gu/Zn SOD), and *SOD2* (Fe/Mn SOD)), and one downregulated superoxide dismutase (*SOD1* (Gu/Zn SOD)). SOD is a metal enzyme. Salt stress induced the expression of Gu/Zn SOD and Fe/Mn SOD genes, which is similar to the expression of antioxidant enzyme genes in *Lycopersicon esculentum* and *Vigna radiata* (Guan et al., 2017; Zhu et al., 2021). CAT is an important antioxidant enzyme system. For example, in rice, the expression of *CATA* and *CATC* is controlled by circadian rhythm. Under salt stress, *CATC* regulates H_2_O_2_ homeostasis and improves salt tolerance in rice (Tounsi et al., 2019). In *Chenopodium quinoa* willd., 4 genes of the CAT family are upregulated to deal with the damage caused by salt stress. This coincides with the findings from the current investigation on the expression of the two genes known to influence CAT activity. The above results show that some important biological activities are related to the transcriptional expression of proline, SOD, and CAT genes that regulate plant salt tolerance (Sun et al., 2021).

### Main genes affecting salt tolerance of *G. maritima* seedlings

In addition to participating in the antioxidant process, *G. maritima’s* tolerance to salt is influenced by changes in the amount of the plant hormone ABA and the MAPK signaling pathway. Responses to salt stress are regulated in part by plant hormones (Ryu and Cho, 2015) A survey of genes involved in hormone signaling pathways has shown that they have a role in *G. maritima’s* salt tolerance. Several plant species have had their salt tolerance increased by evidence showing that ABA and JA were present (Goossens et al., 2016; Vishwakarma et al., 2017; Yang et al., 2017). In plants, ABA plays a crucial role as a regulator of signal transduction processes (Sicilia et al., 2019). As a result of stimulus, ABA-bound PYR/PYL/RCAR inhibits PP2Cs, activating SnRK2. As a double-negative regulator, this protein complex raises the expression of linked genes in response to ABA (Huang et al., 2018). NaCl stress was shown to cause an increase in the expression of the ABF gene in *G. maritima* via downregulating the activity of SnRK2. Previous studies on plants have shown the same results, thus these results are reliable (Yang et al., 2017; Lei et al., 2018; Lei et al., 2021). Numerous studies have revealed that exogenous ABA administration in plants activates the MAPK pathway, which is then responsible for transcriptional regulation. This suggests that the MAPK pathway plays an important role in ABA signal transduction (Zhang et al., 2014; Li et al., 2016; Li et al., 2017). The MEK1-MKK2-MPK6/MPK4 pathway improves salt tolerance in Arabidopsis via an interaction between MKK2 and MPK4 (Teige et al., 2004). The MAPK cascade MEKK1-MKK2-MPK6/MPK4 plays a critical role in regulating ROS balance and stress signal transmission during salt stress by responding to SA and rosin (Yang and Guo, 2018a). After 4 hours of exposure to salt stress, cotton’s MAPK cascade gene expression is considerably upregulated (Chen et al., 2020). The initial step in ABA signal transduction involves the PYR/PYL/RCAR protein and phosphatase/kinase pairs PP2Cs and SnRK2s, respectively, with antagonistic functions. ABA signal transduction results in transcription factors activating gene expression under the control of SnRK2s (Cutler et al., 2010; Finkelstein, 2013; Tan et al., 2018). In the present study, after *G. maritima* was subjected to salt stress, PP2C was upregulated and inhibited the activity of SnRK2 kinase. The expression of *MAPKK17-18* in the downstream MAPK signaling pathway was promoted, alleviating leaf senescence and enhancing the salt tolerance of *G. maritima.* This is similar to the findings of a previous study on *Arabidopsis* (Matsuoka et al., 2015).

In addition to regulating the SOD and CAT processes, glutathione metabolism is an important regulator. Oxidized glutathione (GSSG) is converted back to glutathione by GR catalysis (GSH) (Tao et al., 2003). Neto showed that GR activity in plant leaves increases significantly after salt stress treatment compared with that in the control (De et al., 2006). Huang found that tomato seedlings under 80 mM salt treatment resist salt stress by significantly enhancing the activities of GR, MDHAR, APX, and DHAR in chloroplasts (Huang and Shan, 2018). Under high salt stress, an increase in plant ROS leads to cell membrane lipid peroxidation. The level of MDA indicates the extent to which plant membranes have been damaged by salt stress. MDA is the primary byproduct of membrane lipid peroxidation. Valentina discovered that in salt-tolerant tomato plants, GSH production and accumulation are triggered by salt stress. Additionally, significant amounts of GSH, GPX, GR, and GSSG are present in salt-tolerant tomato, which helps to reduce membrane lipid peroxidation due to salt stress (Mittova et al., 2003). Analysis of transcriptome data showed that modulation of eight genes involved in glutathione metabolism was a major mechanism impacting *G. maritima’s* salt tolerance. These genes are involved in regulating the activity of GR and the synthesis of GSH. After being put through the ringer by biological and abiotic stresses, plants create flavonoids and flavonoids to eliminate H_2_O_2_, boost plant antioxidant activity, minimize water loss, improve the environment in plant cells, and resist damage, as proven in several studies (Nakabayashi et al., 2014). However, by transcriptome analysis of *G. maritima*, the three genes involved in flavonoid, flavone, and flavonol biosynthesis, 4,5,6,7-trihydroxyflavonol, quercetin, and myricetin, were found to be downregulated. A study also showed that sea milkweed does not improve its antioxidant activity through flavonoids after salt stress.

## Conclusion

In this study, *G. maritima* was a salt secreting plant, and the salt secreting tissue in its leaves was salt glands, which sank into epidermal cells and consisted of stem cells, collecting cells, and secreting cells. After high salt stress, the leaf cells of *G. maritima* grass show a decrease in volume and become densely arranged. An increase in salt concentration will increase the water loss effect of the leaves. After high salt stress, a large amount of proline is produced in the leaves to regulate osmotic balance, and the activities of SOD and POD enzymes in the antioxidant enzyme system are significantly increased to eliminate the large amount of ROS generated due to salt damage, ensuring normal growth in a high salt environment. A study on the molecular mechanism of sea milkweed leaves revealed 11 DEGs that regulate proline synthesis and CAT and SOD activities, which may be key genes affecting salt tolerance of *G. maritima*. It is also located in Plant hormone signaling, Glutathione metabolism and MAPK signaling pathway-plant. The KEGG pathway, including flavonoid biosynthesis, flavonoid biosynthesis, and flavonol biosynthesis, may also be important metabolic pathways that affect salt tolerance of *G. maritima*. Studying and analyzing 93 DEGs in these pathways can lay the foundation for further exploring salt tolerance genes and improving salt tolerance in forage crops. It also provides precious biological improvement resources for improving Alkali soil.

## Data availability statement

The datasets presented in this study can be found in online repositories. The names of the repository/repositories and accession number(s) can be found in the article/**Supplementary Material**.

## Author contributions

FS designed the experiment, RG, ZW, XL and YY performed the experiments, RG and FT analyzed the data, RG wrote the manuscript. All authors reviewed the manuscript. All authors contributed to the article and approved the submitted version.
