# Supplementary material for: Physiological and transcriptomic analysis of salt tolerant Glaux maritima grown under high saline condition
Source: Front Plant Sci. 2023 Aug 29;14:1173191. doi: 10.3389/fpls.2023.1173191 (PMC10497109; doi:10.3389/fpls.2023.1173191)
Supplement: Supplementary file 1 [file DataSheet_1.docx]

**Attached Figure**

**
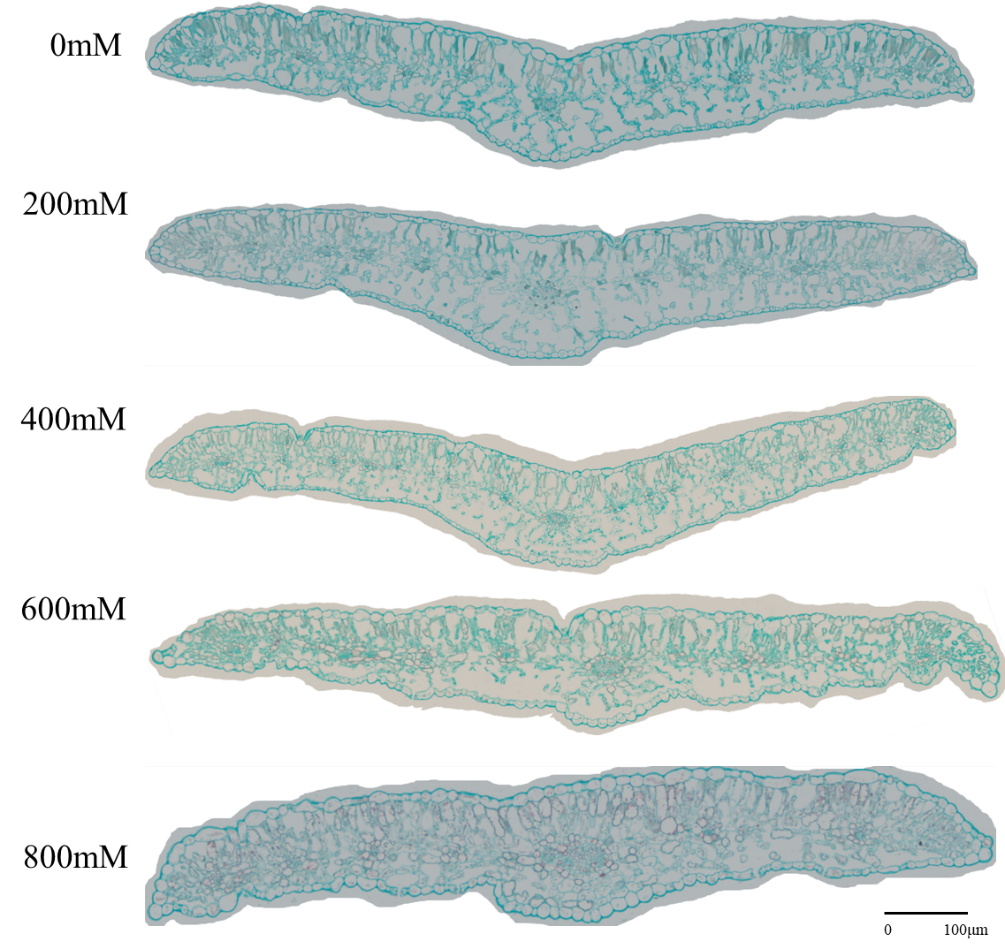
**

Attached Figure1 Cross section of *G. maritima* leaves under different salt concentrations

**Supplementary Table**

Supplementary Table 1. the quality of the isolated RNA samples

| number | Sample name | Concentration (ng/ul) | Volume (ul) | Total(ug) |
| --- | --- | --- | --- | --- |
| 1 | N0-1 | 258 | 33 | 8.51 |
| 2 | N0-2 | 255 | 33 | 8.42 |
| 3 | N0-3 | 296 | 33 | 9.77 |
| 4 | N3-1 | 357 | 33 | 11.78 |
| 5 | N3-2 | 303 | 33 | 10 |
| 6 | N3-3 | 284 | 33 | 9.37 |
| 7 | N4-1 | 228 | 33 | 7.52 |
| 8 | N4-2 | 387 | 33 | 12.77 |
| 9 | N4-3 | 173 | 33 | 5.71 |

Note: N0:0mM/L NaCl stress; N3:600mM/L NaCl stress; N4:800mM/L NaCl stress.

**
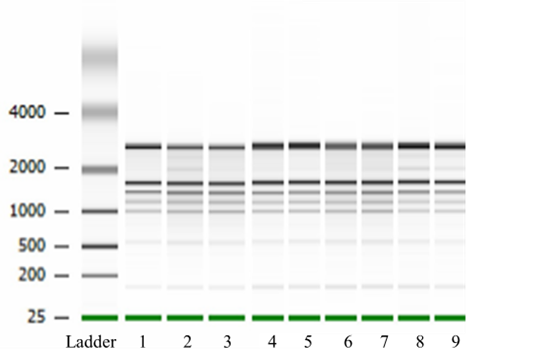
**

Attached Figure2 Agilent 2100 RNA 6000 Nano kit detection

Note:1-3: N0:0mM/L NaCl stress;4-6: 600mM/L NaCl stress;7-9: 800mM/L NaCl stress;

Supplementary Table2. Sequencing data of *G. maritima* leaves under different salt concentrations

| Concentrations | Sequencing data |
| --- | --- |
| N0-1 | 8.48G |
| N0-2 | 7.17G |
| N0-3 | 8.27G |
| N3-1 | 8.36G |
| N3-2 | 6.82G |
| N3-3 | 7.99G |
| N4-1 | 6.31G |
| N4-2 | 6.45G |
| N4-3 | 6.23G |

Note: N0:0mM/L NaCl stress; N3:600mM/L NaCl stress; N4:800mM/L NaCl stress.

Supplementary Table3. Statistics on differentially expressed genes in three upregulated KEGG enrichment pathways (glutathione metabolism, and plant hormone signal transduction, MAPK signaling pathway-plant)

| Gene-ID | Symbol | N0 | N3 | N4 | Profile | Pathway |
| --- | --- | --- | --- | --- | --- | --- |
| Unigene0025663 | *GSH1* | 0 | 2.08 | 3.01 | 7 | Glutathione metabolism |
| Unigene0037990 | *At3g24170* | 0 | 0.68 | 1.29 | 7 | Glutathione metabolism |
| Unigene0041171 | *ODC* | 0 | 2.83 | 4.21 | 7 | Glutathione metabolism |
| Unigene0042461 | *PGD3* | 0 | 0.66 | 1.27 | 7 | Glutathione metabolism |
| Unigene0043960 | *APIC* | 0 | 1.61 | 2.25 | 7 | Glutathione metabolism |
| Unigene0049105 | *GSTU7* | 0 | 0.97 | 1.77 | 7 | Glutathione metabolism |
| Unigene0061720 | *GOR* | 0 | 0.64 | 1.56 | 7 | Glutathione metabolism |
| Unigene0070188 | *GSTU17* | 0 | 1.89 | 3.59 | 7 | Glutathione metabolism |
| Unigene0003935 | *WRKY22* | 0 | 3.31 | 1.84 | 6 | MAPK signaling pathway - plant |
| Unigene0060296 | *NPK1* | 0 | 1.72 | 1.34 | 6 | MAPK signaling pathway - plant |
| Unigene0050694 | *HMA5* | 0 | 1.35 | 1.15 | 6 | MAPK signaling pathway - plant |
| Unigene0057791 | *CAT3* | 0 | 1.17 | 1.37 | 6 | MAPK signaling pathway - plant |
| Unigene0031037 | *MKK9* | 0 | 1.49 | 1.97 | 6 | MAPK signaling pathway - plant |
| Unigene0061713 | *VIP1* | 0 | 2.08 | 2.4 | 6 | MAPK signaling pathway - plant |
| Unigene0030381 | *MAPKKK18* | 0 | 3.04 | 4.39 | 7 | MAPK signaling pathway - plant |
| Unigene0070986 | *MAPKKK18* | 0 | 2.36 | 3 | 6 | MAPK signaling pathway - plant |
| Unigene0002691 | *CAT1* | 0 | 1.75 | 2.49 | 7 | MAPK signaling pathway - plant |
| Unigene0070829 | *MPK9* | 0 | 1.04 | 0.88 | 6 | MAPK signaling pathway - plant |
| Unigene0076711 | *HMA4* | 0 | 0.74 | 1.21 | 7 | MAPK signaling pathway - plant |
| Unigene0002108 | *TIR1* | 0 | 1.14 | 1.45 | 6 | Plant hormone signal transduction |
| Unigene0009059 | *LAX2* | 0 | 1.17 | 1.49 | 6 | Plant hormone signal transduction |
| Unigene0007479 | *ABF4* | 0 | 2.22 | 1.82 | 6 | Plant hormone signal transduction |
| Unigene0007940 | *SAUR50* | 0 | 3.21 | 3.08 | 6 | Plant hormone signal transduction |
| Unigene0005652 | *AHK3* | 0 | 0.99 | 1.23 | 6 | Plant hormone signal transduction |
| Unigene0021538 | *At2g29380* | 0 | 4.06 | 3.3 | 6 | Plant hormone signal transduction |
| Unigene0025150 | DPBF3 | 0 | 1.25 | 0.93 | 6 | Plant hormone signal transduction |
| Unigene0034631 | XTH24 | 0 | 6.59 | 7.88 | 6 | Plant hormone signal transduction |
| Unigene0022367 | *COI1* | 0 | 1.13 | 0.96 | 6 | Plant hormone signal transduction |
| Unigene0026327 | *IAA26* | 0 | 3.21 | 3.61 | 6 | Plant hormone signal transduction |
| Unigene0034253 | *AHP5* | 0 | 0.9 | 1.1 | 6 | Plant hormone signal transduction |
| Unigene0023192 | *BSK1* | 0 | 1.02 | 1.12 | 6 | Plant hormone signal transduction |
| Unigene0043863 | *JAR4* | 0 | 1.17 | 1.19 | 6 | Plant hormone signal transduction |
| Unigene0044134 | *IAA7* | 0 | 1.65 | 1.22 | 6 | Plant hormone signal transduction |
| Unigene0052637 | *IAA1* | 0 | 1.19 | 1.08 | 6 | Plant hormone signal transduction |
| Unigene0055112 | *IAA26* | 0 | 1.52 | 1.42 | 6 | Plant hormone signal transduction |
| Unigene0055113 | *IAA26* | 0 | 1.59 | 1.85 | 6 | Plant hormone signal transduction |
| Unigene0055114 | *IAA26* | 0 | 2.06 | 2.31 | 6 | Plant hormone signal transduction |
| Unigene0064363 | *BKI1* | 0 | 1.25 | 0.8 | 6 | Plant hormone signal transduction |
| Unigene0070830 | *TGA4* | 0 | 1.28 | 1.36 | 6 | Plant hormone signal transduction |
| Unigene0073747 | *COI1* | 0 | 1.25 | 1.11 | 6 | Plant hormone signal transduction |
| Unigene0074764 | *ABI5* | 0 | 6.09 | 5.14 | 6 | Plant hormone signal transduction |
| Unigene0078257 | *ABF2* | 0 | 4.19 | 3.91 | 6 | Plant hormone signal transduction |
| Unigene0078711 | *ABI5* | 0 | 12.55 | 11.54 | 6 | Plant hormone signal transduction |
| Unigene0003217 | *PP2CA* | 0 | 1.69 | 1.37 | 6 | Plant hormone signal transduction; |
| Unigene0003302 | *ABF2* | 0 | 4.18 | 3.73 | 6 | Plant hormone signal transduction; MAPK signaling pathway - plant |
| Unigene0006381 | *HAB2* | 0 | 3.47 | 3.69 | 6 | Plant hormone signal transduction; MAPK signaling pathway - plant |
| Unigene0007729 | *PYL3* | 0 | 1.76 | 1.67 | 6 | Plant hormone signal transduction; MAPK signaling pathway - plant |
| Unigene0008867 | *SRK2E* | 0 | 1.06 | 0.82 | 6 | Plant hormone signal transduction; MAPK signaling pathway - plant |
| Unigene0021981 | *AHG1* | 0 | 3.62 | 4.34 | 6 | Plant hormone signal transduction; MAPK signaling pathway - plant |
| Unigene0022574 | *ABI1* | 0 | 3.5 | 2.8 | 6 | Plant hormone signal transduction; MAPK signaling pathway - plant |
| Unigene0031128 | *SAPK10* | 0 | 1.26 | 1.34 | 6 | Plant hormone signal transduction; MAPK signaling pathway - plant |
| Unigene0038049 | *EBF2* | 0 | 4.84 | 4.62 | 6 | Plant hormone signal transduction; MAPK signaling pathway - plant |
| Unigene0038050 | *EBF1* | 0 | 2.28 | 2.3 | 6 | Plant hormone signal transduction; MAPK signaling pathway - plant |
| Unigene0040625 | *PYL3* | 0 | 1.72 | 1.6 | 6 | Plant hormone signal transduction; MAPK signaling pathway - plant |
| Unigene0046708 | *ERF.C.3* | 0 | 3.17 | 3.06 | 6 | Plant hormone signal transduction; MAPK signaling pathway - plant |
| Unigene0068779 | *HAB1* | 0 | 1.95 | 1.89 | 6 | Plant hormone signal transduction; MAPK signaling pathway - plant |
| Unigene0068862 | *ETR2* | 0 | 1.51 | 1.51 | 6 | Plant hormone signal transduction; MAPK signaling pathway - plant |
| Unigene0068974 | *MEKK1* | 0 | 1.54 | 1.52 | 6 | Plant hormone signal transduction; MAPK signaling pathway - plant |
| Unigene0070955 | *PP2C06* | 0 | 4.43 | 4.61 | 6 | Plant hormone signal transduction; MAPK signaling pathway - plant |
| Unigene0071276 | *EIN3* | 0 | 1.62 | 1.5 | 6 | Plant hormone signal transduction; MAPK signaling pathway - plant |
| Unigene0071511 | *HAB1* | 0 | 4.31 | 3.59 | 6 | Plant hormone signal transduction; MAPK signaling pathway - plant |
| Unigene0073615 | *CTR1* | 0 | 1.43 | 1.37 | 6 | Plant hormone signal transduction; MAPK signaling pathway - plant |
| Unigene0077023 | *MYC2* | 0 | 1.33 | 1.44 | 6 | Plant hormone signal transduction; MAPK signaling pathway - plant |
| Unigene0078774 | *EBF2* | 0 | 3.5 | 3.79 | 6 | Plant hormone signal transduction; MAPK signaling pathway - plant |
| Unigene0006382 | *PP2C06* | 0 | 2.08 | 3.06 | 7 | Plant hormone signal transduction; MAPK signaling pathway - plant |
| Unigene0028951 | *ETR2* | 0 | 2.51 | 3.52 | 7 | Plant hormone signal transduction; MAPK signaling pathway - plant |
| Unigene0046695 | *SAPK2* | 0 | 0.95 | 1.52 | 7 | Plant hormone signal transduction; MAPK signaling pathway - plant |
| Unigene0072371 | *SRK2A* | 0 | 0.48 | 1.66 | 7 | Plant hormone signal transduction; MAPK signaling pathway - plant |

Note: N0:0mM/L NaCl stress; N3:600mM/L NaCl stress; N4:800mM/L NaCl stress.

Supplementary Table4. provides statistics on the three KEGG enrichment pathways that have had their expression levels downregulated (flavone and flavonol biosynthesis pathway and flavonoid biosynthesis pathway)

| Gene ID | N0 | N3 | N4 | Symbol | Profile | Pathway |
| --- | --- | --- | --- | --- | --- | --- |
| Unigene0036667 | 0 | -1.95 | -2.88 | *CYP75A1* | 0 | Flavone and flavonol biosynthesis |
| Unigene0058443 | 0 | -1.46 | -2.07 | *CYP75B2* | 0 | Flavonoid biosynthesis; Flavone and flavonol biosynthesis |
| Unigene0002287 | 0 | -5.88 | -4.86 | *CCOMT* | 1 | Flavonoid biosynthesis |
| Unigene0002631 | 0 | -4.42 | -2.8 | *CHS1* | 1 | Flavonoid biosynthesis |
| Unigene0006015 | 0 | -2.12 | -1.55 | *CHI3* | 1 | Flavonoid biosynthesis |
| Unigene0014653 | 0 | -3.14 | -2.4 | *ANR* | 1 | Flavonoid biosynthesis |
| Unigene0014654 | 0 | -2.87 | -3 | *ANR* | 1 | Flavonoid biosynthesis |
| Unigene0016449 | 0 | -4.3 | -2.73 | *CHS1* | 1 | Flavonoid biosynthesis |
| Unigene0027021 | 0 | -5.29 | -3.32 | *CYP75A1* | 1 | Flavonoid biosynthesis; Flavone and flavonol biosynthesis |
| Unigene0032781 | 0 | -5.52 | -4.98 | *CHS1* | 1 | Flavonoid biosynthesis |
| Unigene0032782 | 0 | -3.32 | -1.96 | *CHS2* | 1 | Flavonoid biosynthesis |
| Unigene0032785 | 0 | -4.87 | -3.13 | *CHS1* | 1 | Flavonoid biosynthesis |
| Unigene0034459 | 0 | -3.59 | -1.83 | *DFR* | 1 | Flavonoid biosynthesis |
| Unigene0040334 | 0 | -1.25 | -0.86 | *CHS2* | 1 | Flavonoid biosynthesis |
| Unigene0042054 | 0 | -1.43 | -1.28 | *CHS* | 1 | Flavonoid biosynthesis |
| Unigene0048785 | 0 | -3.54 | -2.65 | *SALAT* | 1 | Flavonoid biosynthesis |
| Unigene0053664 | 0 | -4.58 | -2.76 | *LDOX* | 1 | Flavonoid biosynthesis |
| Unigene0053665 | 0 | -5.91 | -3.14 | *LDOX* | 1 | Flavonoid biosynthesis |
| Unigene0054733 | 0 | -3.96 | -2.74 | *F3H-2* | 1 | Flavonoid biosynthesis |
| Unigene0056362 | 0 | -5.31 | -3 | *--* | 1 | Flavonoid biosynthesis |
| Unigene0056363 | 0 | -4.99 | -3.61 | *LAR* | 1 | Flavonoid biosynthesis |
| Unigene0059333 | 0 | -4.24 | -3.34 | *CHS* | 1 | Flavonoid biosynthesis |
| Unigene0069098 | 0 | -5.33 | -3.44 | *DFR* | 1 | Flavonoid biosynthesis |
| Unigene0071330 | 0 | -1.48 | -1.52 | *CHI* | 1 | Flavonoid biosynthesis |
| Unigene0073572 | 0 | -5.69 | -3.74 | *LAR* | 1 | Flavonoid biosynthesis |
| Unigene0073877 | 0 | -1.35 | -0.78 | *CHS2* | 1 | Flavonoid biosynthesis |

Note: N0:0mM/L NaCl stress; N3:600mM/L NaCl stress; N4:800mM/L NaCl stress.

Supplementary Table 5. Fluorescent quantitative primer sequences

| Gene | Primer sequence | Fragment size |
| --- | --- | --- |
| β-Actin -F | TGGCACTTGATTACGAGCAG | 160bp |
| β-Actin -R | GGAGCTTCCATTCCAATCAA |  |
| Unigene0016449-F | TCCGAGATCACTGCTGTCAC | 186bp |
| Unigene0016449-R | CCCATCACTATCCGGAAGAA |  |
| Unigene0040334-F | TTGGGGAAAGATGTGCCTAC | 194bp |
| Unigene0040334-R | AGCACTTCTCGAGTCGCTTC |  |
| Unigene0056362-F | GGTCGAGTTCTCATCATTGGA | 162bp |
| Unigene0056362-R | GATAGCGCCTTTGTCTTGGA |  |
| Unigene0069098-F | TGAGAGGAATGGGATTCGAG | 122bp |
| Unigene0069098-R | GCCATTGTCCTTAGCAGGAG |  |
| Unigene0071330-F | TCACCGCCTTAAAAATCGAG | 198bp |
| Unigene0071330-R | CACTCTTCCCCTTCCACTTG |  |
| Unigene0073877-F | TCTCGAGCAAAATCGAGGAT | 141bp |
| Unigene0073877-R | CTCCGGTCAAGTTCAGCTTC |  |
| Unigene0072782-F | GAACCAAAGAAGGCAATCCA | 115bp |
| Unigene0072782-R | TTGGATGGAGGGCAGATAAG |  |
| Unigene0002108-F | CCTGATAATTGGGGAGCTGA | 114bp |
| Unigene0002108-R | GCACAAACTCTCATCGGTCA |  |
| Unigene0008867-F | GGAAGGTTCAATGAGGACGA | 189bp |
| Unigene0008867-R | TTGCGAGTGCAGTACCAAAG |  |
| Unigene0009059-F | GTGGAAACCCCAGAAGTTCA | 179bp |
| Unigene0009059-R | GCATGAGGATAACAGCAGCA |  |
| Unigene0031128-F | GCCATGGTGTGCATTTTGTA | 169bp |
| Unigene0031128-R | GAGCAGGACTTCCATCAAGC |  |
| Unigene0068862-F | GAAGGTTTTTGGAACGTGGA | 120bp |
| Unigene0068862-R | CACGTTCGAGCAACTCAAAA |  |
| Unigene0070829-F | GCATATTTGCGGAATTGCTT | 194bp |
| Unigene0070829-R | TGGGAACTTTTGGGTGAAAG |  |
| Unigene0073615-F | TTTCTACCCCATTGCGTTTC | 153bp |
| Unigene0073615-R | CAGCAGCATCTCCATCAAAA |  |
| Unigene0002691-F | TAGGGATCCTCGTGGTTTTG | 101bp |
| Unigene0002691-R | TCATTCCATCACGAACGAAA |  |
| Unigene0025663-F | CTCCCTTGGCTTGGATATGA | 101bp |
| Unigene0025663-R | CAAGACCAGCACGGAATTTT |  |
| Unigene0037990-F | GAAAAATTTCCGGTCCCAAT | 126bp |
| Unigene0037990-R | CTTGGCCAGGAATATGAGGA |  |
| Unigene0070188-F | CGGCAAAAGATGAAGAGGAG | 158bp |
| Unigene0070188-R | ACCCAAGTAGCACCCAAGTG |  |
